# Fault Diagnosis for High-Speed Train Axle-Box Bearing Using Simplified Shallow Information Fusion Convolutional Neural Network

**DOI:** 10.3390/s20174930

**Published:** 2020-08-31

**Authors:** Honglin Luo, Lin Bo, Chang Peng, Dongming Hou

**Affiliations:** 1 The State Key Laboratory of Mechanical Transmission, Chongqing University, Chongqing 400044, China; 20097477@cqu.edu.cn; 2 National Engineering Laboratory for High-Speed Train, CRRC Qingdao Sifang Co. Ltd., Qingdao 266000, China; pengchang1234@126.com; 3 School of Mechanical, Electronic and Control Engineering, Beijing Jiaotong University, Beijing 100044, China; 18116010@bjtu.edu.cn

**Keywords:** axle-box bearing, convolutional neural network, simplified shallow information fusion CNN, fault diagnosis

## Abstract

Axle-box bearings are one of the most critical mechanical components of the high-speed train. Vibration signals collected from axle-box bearings are usually nonlinear and nonstationary, caused by the complicated operating conditions. Due to the high reliability and real-time requirement of axle-box bearing fault diagnosis for high-speed trains, the accuracy and efficiency of the bearing fault diagnosis method based on deep learning needs to be enhanced. To identify the axle-box bearing fault accurately and quickly, a novel approach is proposed in this paper using a simplified shallow information fusion-convolutional neural network (SSIF-CNN). Firstly, the time domain and frequency domain features were extracted from the training samples and testing samples before been inputted into the SSIF-CNN model. Secondly, the feature maps obtained from each hidden layer were transformed into a corresponding feature sequence by the global convolution operation. Finally, those feature sequences obtained from different layers were concatenated into one-dimensional as the fully connected layer to achieve the fault identification task. The experimental results showed that the SSIF-CNN effectively compressed the training time and improved the fault diagnosis accuracy compared with a general CNN.

## 1. Introduction

Axle-box bearings, as the key component of the high-speed train, can have a significant impact on the security, stability and sustainability of railway vehicles [1]. If an axle-box bearing failure is not detected promptly, it may cause severe delays or even dangerous derailments, implicating human life prejudice and significant costs for railway managers and operators. Therefore, how to identify the axle-box bearing fault accurately and quickly has become an urgent challenge to be solved. Currently, vibration analysis, acoustic analysis and temperature analysis are three main approaches for axle-box bearings failure detection [2]. However, the temperature does not raise much for an early-stage bearing fault, and noises from wheel/rail contacts and the train drive system, as well as aerodynamic forces, may contaminate the signal acquired by the acoustic arrays [3]. Due to its higher reliability, various fault diagnosis techniques based on vibration signal-processing techniques have been applied to maintain axle-box bearings operating properly and reliably [4,5,6,7]. However, it is a time-consuming and labor-intensive task to determine the type of bearing defects using conventional diagnosis methods based on various signal-processing techniques, which can only achieve a qualitative result without the severity of a bearing fault. Compared with the conventional signal-processing technique-based method, the intelligent diagnosis method based on machine learning can automatically process the bearing data and evaluate the bearing health status comprehensively [8,9].

Taking the advantages in the qualitative and quantitative analyses of bearing faults, various typical intelligent diagnosis techniques have been proposed to improve the bearing fault diagnosis performance. Among them, artificial neural networks (ANN) and support vector machines (SVM) are commonly applied in the detection and diagnosis of machine faults [8,9,10]. Generally, two essential steps are necessary for rotating machinery fault diagnosis based on ANN or SVM: feature extraction using signal-processing techniques and fault classification using pattern recognition techniques. Jamadar et al. [11] adopted a back propagation neural network (BPNN) to classify various bearing faults using 24 dimensionless parameters. Van et al. [12] proposed a novel wavelet kernel function-based SVM model for bearing fault diagnosis using features extracted from the signals, which are decomposed by nonlocal means (NLM) and empirical mode decomposition (EMD). Chen et al. [13] applied ANN for diagnosing bearing fault severity using the simulation data to solve the issue of adequate data requirement of the ANN. Lei et al. [14] employed the ensemble empirical mode decomposition (EEMD) for feature extraction and wavelet neural network (WNN) for diagnosing bearing faults. Tang et al. [15] carried out SVM to process the Shannon entropy and autoregressive model coefficients for detecting the different faults. Batista et al. [16] combined different SVMs to detect the bearing failures using 13 statistical parameters in the time domain and frequency domain. In addition, some improved methods, such as hidden markov, adaptive neuro-fuzzy inference system (ANFIS), extreme learning machine (ELM) and support tensor machine (STM), have been proposed to implement the bearing fault diagnosis and classification [17,18,19,20].

Although traditional intelligent diagnosis methods have been widely applied in machinery fault diagnosis, they still have three inherent disadvantages [8,21]: (1) Signal feature extraction is easily affected by the complicated working conditions, and the signal features of composite faults cannot be extracted effectively. (2) The sensitive features selected mainly depend on the engineering experience of diagnostic experts. (3) Traditional intelligent diagnosis methods, such as ANN and SVM, belong to shallow learning models, which are difficult to learn complex nonlinear relationships effectively.

Deep learning, as a new machine-learning method, can automatically learn in-depth local features from the raw data for classification to overcome the inherent disadvantages of traditional intelligent methods [10]. The convolution neural network, proposed by LeCun [22,23], is an effective deep-learning method and has been applied in bearing fault diagnosis. Shao et al. [6] proposed a new deep-learning model that combines the advantages of the deep belief network (DBN) and convolutional neural network (CNN) to detect the bearing failure. Lo et al. [24] propose a novel prognostic method based on a 1D CNN with clustering loss by classification training to detect bearing and gear wears. Chen et al. [25] proposed a novel fault diagnosis method integrating CNN and ELM to reduce the training complexity and obtain robust features. Wang et al. [26] proposed a modified fault diagnosis method combining CNN and hidden markov models (HMM) to classify rolling element bearing faults. Janssens et al. [27] proposed a 2D CNN with one convolutional layer to learn useful features extracted from the frequency spectrum using two accelerometers for bearing fault detection. Chen et al. [28] proposed a novel CNN model named the convolution recurrent neural network, which combines the advantages of the CNN and recurrent neural network (RNN) to build up end-to-end health indicators of bearings adaptively. Mao et al. [21] proposed a new method for bearing incipient fault online detection using semi-supervised architecture and deep feature representation. Wang et al. [29] presented a comprehensive survey on deep-learning techniques and their applications in smart manufacturing, in which four typical deep-learning models, including the CNN, restricted Boltzmann machine, auto encoder and RNN, are discussed in detail.

Although the CNN has achieved some results in bearing fault diagnosis, the diagnosis performance of CNN still needs to be improved to meet the requirement of axle-box bearing fault diagnosis. Different from the fault diagnosis for other industrial equipment, the fault diagnosis of railway transportation equipment has its special characteristics. For high-speed trains, safety is the priority. The fault diagnosis model should process the bearing data quickly and accurately to meet stringent reliability and real-time requirements for the failure monitoring of axle-box bearings. Due to the complexity of the CNN, more layers mean more convolution kernels, and each neuron multiplies the input data with connection weights, which will lead to the size of the parameter of the CNN being more than tens or even hundreds of thousands. More computational burdens and longer training times are needed due to the large size of the parameters, which can lead to poorer performance of the CNN. In addition, each layer of the CNN has a different expression of the input data and with multiple layers. However, only the outputs of the last layer are connected to the fully connected layer, and the shallow information in other layers is neglected in a traditional CNN framework. Therefore, it is necessary to reduce the number of model parameters and make use of shallow information to improve the diagnosis performance of the CNN.

Some research has been done to fill this gap. Fu et al. [30] proposed a multiscale comprehensive feature fusion-CNN (MCFF-CNN) based on residual learning for vehicle color recognition and achieved an improved recognition performance. Zhang et al. [31] proposed a compact convolutional neural network augmented with multiscale feature extraction to carry out diagnosis tasks with limited training samples and presented three cases to verify the effectiveness of the proposed method. Meng et al. [32] proposed a CNN-based framework for digital subtraction angiography cerebrovascular segmentation and obtained some results. Jun et al. [33] proposed a multiscale CNN model for bearings’ remaining useful life predictions, in which the last convolutional layer and pooling layer were combined to form a mixed layer before being connected to the fully connected layer. However, the performances of the methods mentioned above still need to be improved.

Aiming to improve the computational efficiency and the diagnostic accuracy, a novel simplified shallow information fusion-convolutional neural network (SSIF-CNN) is proposed for vibration-based axle-box bearing fault diagnosis. The proposed method firstly converts the feature maps obtained from each pooling layer into a feature sequence by the global convolution operation. Then, those feature sequences obtained from different pooling layers are concatenated into a one-dimensional vector before been connected to the classifier through the fully connected layer. The experimental results show that, compared to the traditional CNN, the SSIF-CNN improves the computing efficiency on the premise of ensuring the accuracy of the fault diagnosis.

The contributions of this paper can be summarized as follows:

(a) We employ an SSIF-CNN model structure to extract more identifiable features for the axle-box bearing fault diagnosis. By integrating the simplified shallow information, the features with more information are maintained to enhance the network capacity and to reduce the dimension of the parameter.
(b) Due to fewer fully connected layer parameters in the SSIF-CNN framework, the model computational efficiency and fault diagnosis accuracy are improved.
(c) The proposed systematic approach integrates feature extraction and SSIF-CNN into a framework, which could realize the goal of monitoring axle-box bearing conditions automatically.

The remaining parts of the paper are organized as follows: In Section 2, the modified procedure of the CNN is introduced. In Section 3, the diagnosis procedure using the modified method is proposed. In Section 4, the benchmark data and experimental data are described and analyzed. Finally, some conclusions are presented in Section 5.

## 2. Simplified Shallow Information Fusion CNN

**Figure 1:**
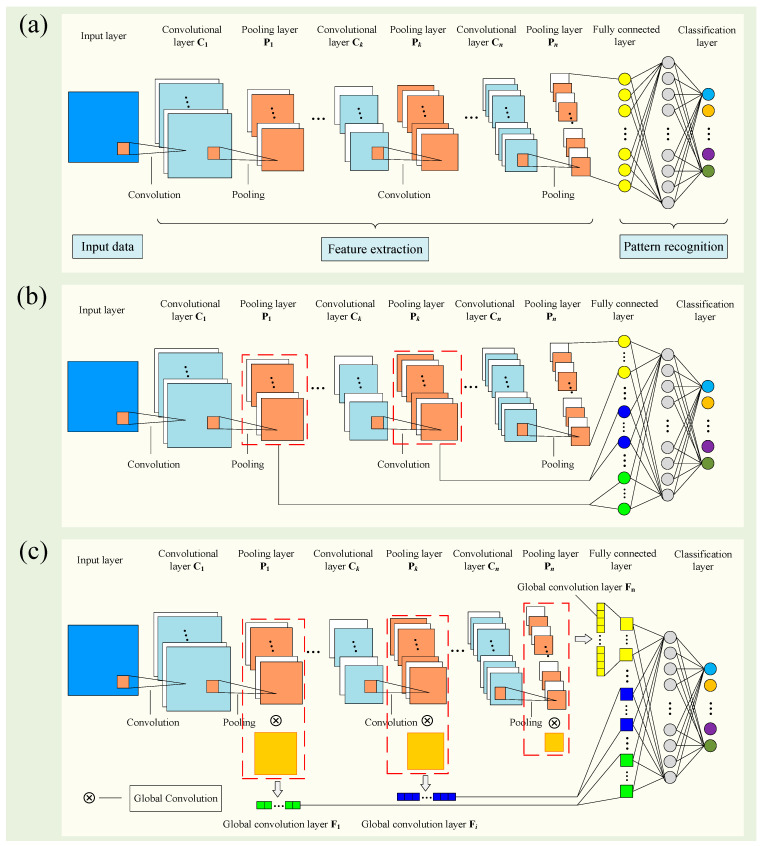
The framework of (**a**) a general convolutional neural network (CNN), (**b**) shallow information fusion-CNN and (**c**) simplified shallow information fusion-CNN.

As shown in Figure 1a, the CNN is a kind of multilayered feedforward neural network, and it mainly contains three parts: a convolutional layers, pooling layers and a fully connected layer. The convolutional layer detects the local conjunctions of the features of the input data by the local convolution operation. The pooling layer merges similar features into one to reduce the size of the network parameters and achieves a translation-invariant characteristic. The fully connected layer converts the inputs into a vector to achieve the categories for different tasks.

In a general CNN architecture, only the outputs of the last layer are connected to the fully connected layer, and the shallow convolution information is neglected. In order to make use of shallow information, the shallow features obtained from shallow pooling layers are connected to the fully connected layer, along with the features of the last layer. As shown in Figure 1b, the yellow circles in the fully connected layer represent the depth information extracted by the last pooling layer$\;P_{n}$, and the blue and green circles represent the shallow information extracted from pooling layer $P_{i}$ and the first pooling layer $P_{1},$ respectively. Each line between the circles represents the connection weight of the neurons. The calculations made by the neurons in the new fully connected layer can be expressed as:

(1) $$fc\left(j\right)=f\left(\sum_{j=1}^{m}\omega_{j}*\left(\sum_{i=1}^{n}P_{i}\right)+b_{j}\right)$$

where fc(j) is the output of the jth neuron in the new fully connected layer,$\;P_{i}=\left(p_{i}^{k},\;k=1,\ldots ,K\right)\;$ is the outputs of the ith pooling layer, K is the number of outputs oft he ith pooling layer, $\omega_{j}$ is the weight vector, $b_{j}\;$is the bias value, *m* is the number of neurons in the new fully connected layer, *n* is the number of pooling layers and f(·) represents the nonlinear activation function. The new fully connected layer contains more neurons due to integrating the shallow information. The shallow information fusion-CNN model has a larger model parameter dimension, which could result in much more computational burdens and longer training times.

In order to reduce the dimension of the model parameters after integrating the shallow information, the feature maps obtained from each pooling layer are transformed into a feature sequence by the global convolution operation before being input into the fully connected layer. As shown in Figure 1c, the global convolution kernels with the same dimension as the feature maps obtained from each pooling layer are used to convolve those corresponding feature maps, and the results extracted from different pooling layers are further concatenated into a 1D feature vector. Then, the 1D feature vector is taken as the new fully connected layer to achieve the pattern recognition task. The green, blue and yellow rectangles represent the feature sequences outputted by using the corresponding global convolution kernels to convolve the outputs of the pooling layer$\;P_{1}$, pooling layer $P_{i}$ and the last pooling layer $P_{n},$ respectively. The global convolution feature sequences obtained from different pooling layers are concatenated as the new fully connected layer before being transmitted to the classification layer. The calculations made by a neuron in the new fully connected layer can be expressed as:

(2) $$fc\left(j\right)=f\left(\sum_{j=1}^{m}\omega_{j}*\left(\sum_{i=1}^{n}\sum_{k=1}^{K}p_{i}^{k}*G_{i}^{k}\right)+b_{j}\right)$$

where fc(j) is the output of the jth neuron in the new fully connected layer,$\;P_{i}=\left(p_{i}^{k},\;k=1,\ldots ,K\right)\;$ is the outputs of the ith pooling layer, K is the number of outputs of the ith pooling layer, $G_{i}^{k}\;$is the corresponding global convolution kernel with the same dimension of$\;P_{i}^{k}$, $\omega_{j}$ is the weight vector, $b_{j}$ is the bias value, *m* is the number of neurons in the new fully connected layer, *n* is the number of pooling layers, f(·) represents the nonlinear activation function and ⊗ represents the global convolution operator.

## 3. Methodology

### 3.1. Axle-Box Bearing Faults Diagnosis Method Based on SSIF-CNN

**Figure 2:**
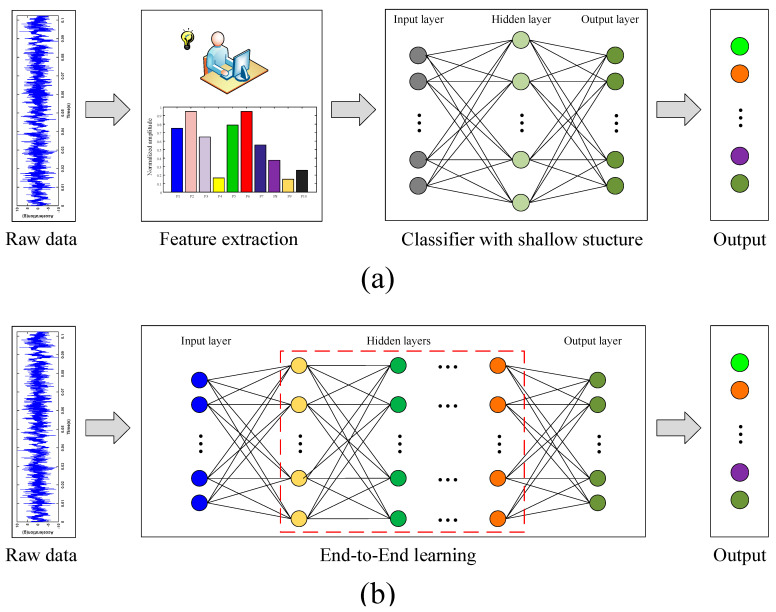
Comparison between two techniques: (**a**) traditional machine learning and (**b**) deep learning.

At present, there are two main technical approaches for machine-learning-based bearing failure diagnosis. As shown in Figure 2a, in the traditional machine-learning-based method, feature extraction and fault classification use different algorithms to achieve the purpose of the final fault classification. On the contrary, in the end-to-end deep-learning-based methods, the two processes of feature extraction and classification can be completed at the same time, as shown in Figure 2b.

**Figure 3:**
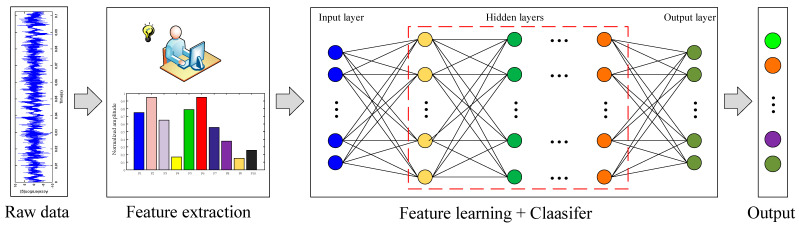
Axle-box bearing fault diagnosis approach based on feature extraction and deep learning.

As shown in Figure 3, the proposed method follows the pattern of feature extraction and deep feature learning rather than the end-to-end learning approach in this work based on the following considerations: (1) Inputting the original vibration signal directly into the CNN model would result in more computational burden and longer training time. (2) Numerous studies have shown that feature extraction based on signal-processing techniques is effective for bearing fault diagnosis. (3) The deep CNN models can learn the local conjunctions of the extracted features without sensitive feature selections.

**Figure 4:**
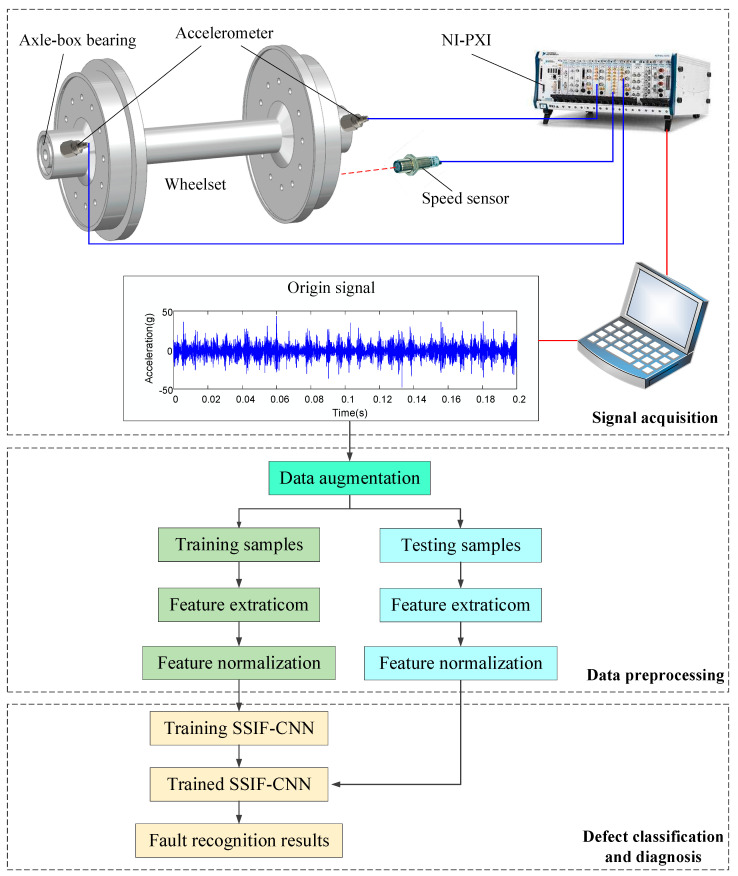
The flowchart of the axle-box bearing faults diagnosis method based on the simplified shallow information fusion-convolutional neural network (SSIF-CNN).

The flowchart of the axle-box bearing faults diagnosis method based on SSIF-CNN is shown in Figure 4. The fault diagnostic process follows the procedure of data acquisition, feature extraction, model training and fault classification.

The algorithm process is illustrated as follows:

- Collect the vibration signals of the axle-box bearing from the acceleration sensors at a particular sampling frequency under various operating conditions.
- Segment the signals to build training samples and testing samples.
- Extract and normalize the features.
- Take the normalized features of the training samples as the input of the diagnosis model and the corresponding fault type as the model label. Use error back propagation to adjust the model. When the error function converges, the model training will be completed.
- Take the normalized features of testing samples as the input of trained model and output the fault recognition results.

### 3.2. Data Augmentation and Feature Extraction

**Figure 5:**
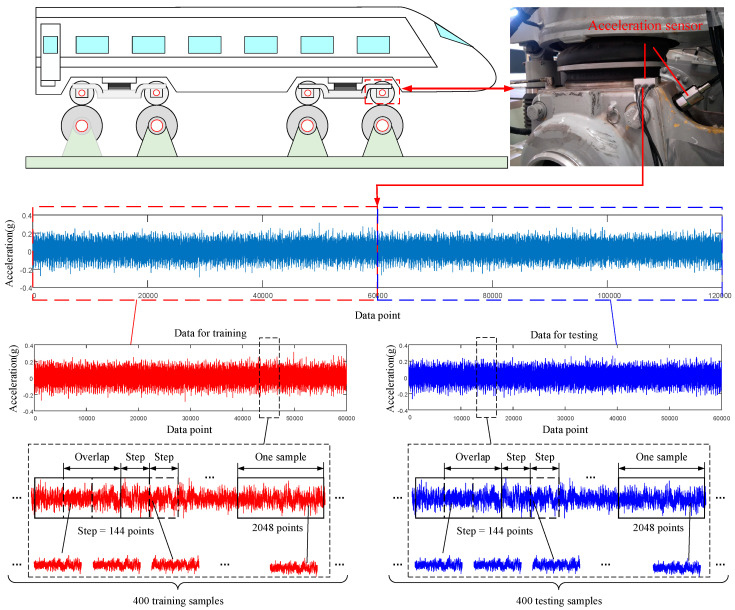
The procedure of generating training and testing samples.

In order to avoid overfitting without sufficient training samples, the data augmentation is essential for improving the generalization and classification accuracy of the CNN. As shown in Figure 5, it is effective to obtain a sufficient number of training samples and testing samples by segmenting overlapping raw data with a specific step length. A vibration signal with 120,000 points can provide 400 training samples and 400 testing samples for the SSIF-CNN when the shift step is 144; the length of each training sample is 2048.

Feature extraction is the first step for bearing fault classification. Time-domain features, which are intuitive and intelligible, constitute the raw data of the bearing running state. Frequency-domain features can describe the variations in the frequency band from the view of the signal spectrum and spectral energy distribution. In total, 29 time and frequency features (P1, P2,…, P29) are calculated in this paper according to reference [8] and reference [20], as illustrated in Table 1.

Since different features have different dimension units, it is necessary to normalize the features and make sure that each feature makes a contribution to the CNN model:

(3) $$P_{norm}=\frac{P-\min\left(P\right)}{\max\left(P\right)-\min\left(P\right)}$$

where P is the feature sequence.

### 3.3. Design of CNNs

When the input data is one-dimensional, the structure of the convolutional kernels in a CNN will be one-dimensional. Considering the limitation of the length and depth of the extracted features, the convolution kernel parameter should not be too large. Since the dimension of the input feature vector, which has 29 feature values, is small, there is no need to pool the output of the convolution layers to reduce the data dimension. The SSIF-CNN used in this work contains only three local convolution layers and three global convolution layers. The specific parameter settings of the three CNN models are shown in Table 2, Table 3 and Table 4.

## 4. Experimental Validation and Verification

In this section, two case studies are carried out to verify the effectiveness of the proposed model. Case 1 focuses on the benchmark data obtained from the Case West Reserve University (CWRU) bearing data center, Cleveland, Ohio, USA. Case 2 is devoted to the axle-box bearing data of the high-speed train collected from the laboratory experiments. The models are implemented on a computer where the CPU is I7-4790-k, the memory is 16 GB and the programming environments are MATLAB R2016 and Python 3.7. The learning rate is 0.01, and maximum number of iterations is 2000.

### 4.1. Numerical Validation

#### 4.1.1. Data Description

The experimental bearing fault datasets that came from the CWRU rolling bearing data center are analyzed to validate the diagnosis performance of the modified model. In this experiment, batches of rolling bearings are processed by electrical discharge machining to simulate different fault types, which include ball fault (BF), inner race fault (IRF) and outer race fault (ORF). The raw vibration datasets, which are obtained from the drive-end bearing under 1797 rpm and sampled at 12 kHz by the accelerometers, are all chosen to recognize the fault patterns. Table 5 shows the information of the benchmark datasets. The depths of the defects are 0.18 mm, 0.36 mm, 0.54 mm and 0.72 mm, while the data of the outer race fault (ORF) with 0.72 mm is not available. More specifications of the rolling element bearings data acquirements can be found on the website [34].

Due to the limited data points of the benchmark data, 120,000 data points were finally picked for each bearing condition in our experiments. Each bearing condition has 400 training samples and 400 testing samples, and each sample contains 2048 data points. The total number of training samples is 4800 (400 × 12) and that of testing samples is also 4800 (400 × 12).

#### 4.1.2. Effect of Sample Size for Training

**Figure 6:**
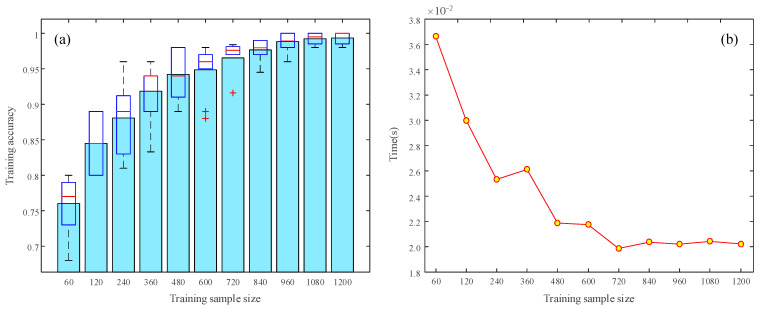
The effect of training sample size on: (**a**) the training accuracy (**b**) time to process one sample.

In order to avoid overfitting and enhance the generalization ability of the SSIF-CNN model, a sufficient number of training samples is needed. Figure 6 shows the effect of the training sample size on the SSIF-CNN performance. To verify the stability of the SSIF-CNN, ten training trials were carried out for each training sample size. The mean value and boxplot of the accuracies of the ten training trials are shown in Figure 6a. As the number of training samples increases, the classification accuracy gradually increases. Even if the training sample size is relatively small, the SSIF-CNN can still achieve a high classification accuracy. Figure 6b shows the average time spent on training by the SSIF-CNN with different sizes of training samples. As the number of training samples increases, the average time required for the SSIF-CNN to process one sample gradually decreases. When the sample size exceeds 840, the modified model only needs about 0.02 s to diagnose a sample, which shows that the SSIF-CNN can meet the real-time requirements of fault diagnosis.

#### 4.1.3. Diagnostic Results

**Figure 7:**
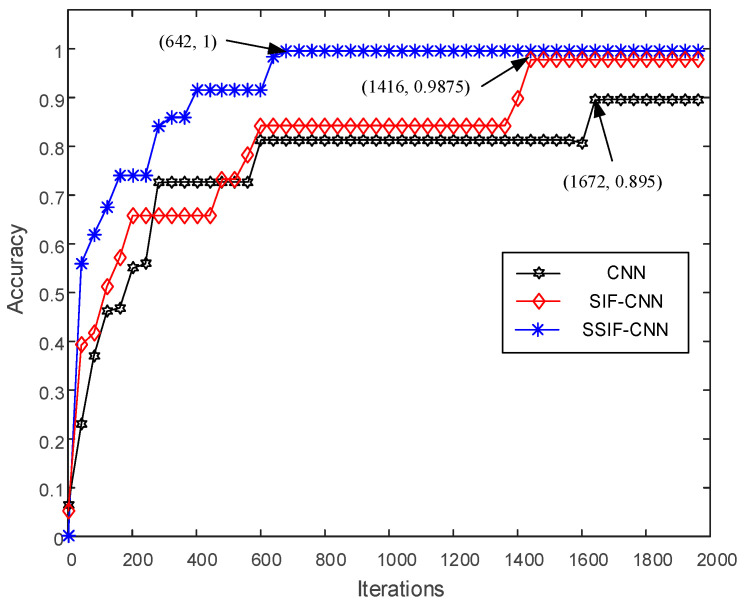
Training accuracy of the CNNs with iterations.

After mixing up the training samples, the whole batch of training samples is input into the training models for ten repeated experiments, and the results of the first trial are shown in Figure 7. As shown in Figure 7, the general CNN will converge after 1672 iterations, with an accuracy at about 89.5%. The accuracy of the SIF-CNN achieves 98.75% after 1416 iterations. However, due to the fewer model parameters, the training accuracy of the SSIF-CNN achieves 100% after only 642 iterations, which is much faster than the general CNN and SIF-CNN.

The fault classification accuracy specifications of the three models are listed in Table 6 and Table 7 in detail. Table 6 shows the specifications of the classification accuracy of the training samples, while Table 7 shows the same thing for the testing samples. In the CNN training process, the accuracy of bearing conditions 7 and 10 only reaches 52% and 42%, whereas all the accuracies of the SIF-CNN model maintain levels above 89%. The SSIF-CNN classifies all the training samples with an accuracy of 100%. In the testing process, the accuracy of bearing conditions 7 and 10 only reaches 31% and 37.5% by the CNN, respectively, and the SIF-CNN model has a classification accuracy with at least 81%. The SSIF-CNN classifies all the training samples with an accuracy of 100%.

**Figure 8:**
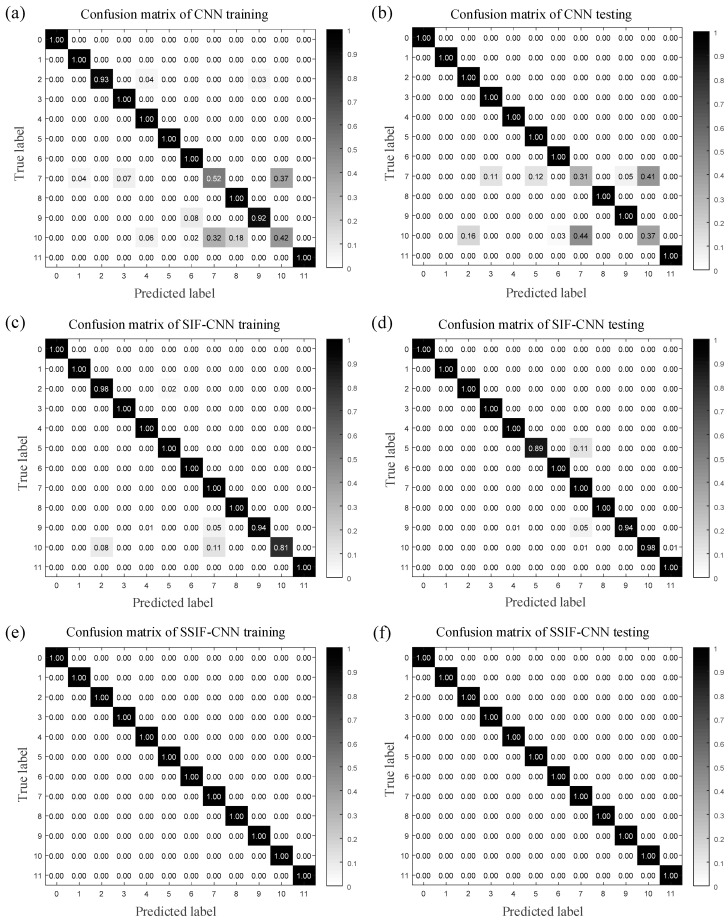
Confusion matrix of the three models for the first trial.

Figure 8 shows how the confusion matrix thoroughly records the diagnosis classification results of the different bearing conditions, including both the classification information and misclassification information. The ordinate axis of the confusion matrix represents the actual label of each bearing condition, and the horizontal axis represents the predicted label. Therefore, the element on the main diagonal of the multiclass confusion matrix represents the diagnosis classification accuracy of each condition. As shown in Figure 8a,b, the CNN fails to classify bearing condition 7 and bearing condition 10. The lowest accuracy happens in condition 10 for training and that of testing happens in condition 7. It can be seen from Figure 8c,d that the lowest accuracy happens in condition 10 for the SIF-CNN training and that of testing happens in condition 5. As shown in Figure 8e,f, the proposed method can classify all the fault types accurately.

**Figure 9:**
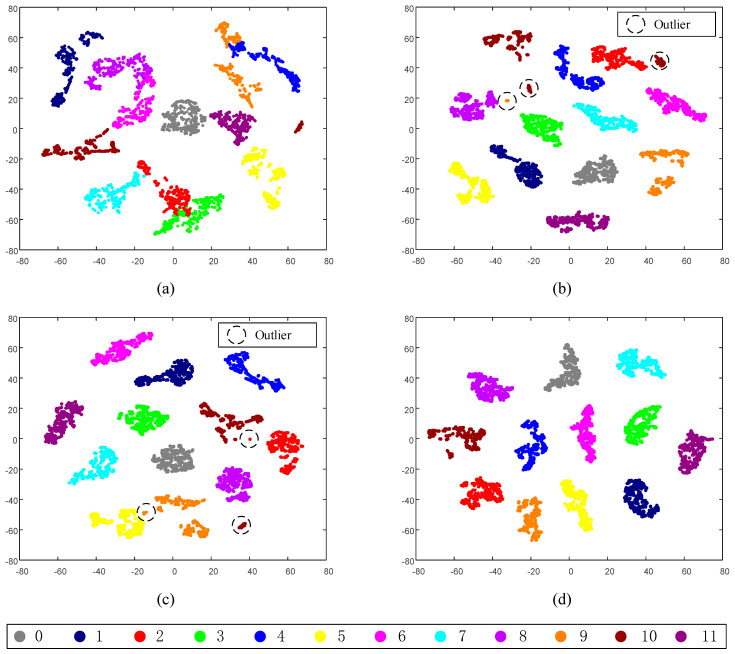
2D visualization of the learned features: (**a**) raw data, (**b**) general CNN, (**c**) SIF-CNN and (**d**) SSIF-CNN.

The *t*-distributed stochastic neighbor embedding [35] (t-SNE) technique is adopted to extract the feature visualizations, and the two-dimensional scatterplot distributions are given in Figure 9. From Figure 9d, the features of different fault types can be clearly classified. The SSIF-CNN can effectively extract features of datasets with different fault categories and different fault depths.

In order to further illustrate the ability of the proposed model in the bearing fault diagnosis, two additional commonly used intelligent methods are applied here as comparative studies. The training samples and testing samples are inputted to the SVM and BPNN. The parameter descriptions of the SVM and BPNN are as follows [25]: (1) SVM: RBF kernel, penal factor equal to 7 and kernel radius equal to 0.1; (2) BPNN: 50 units in the hidden layer; the learning rate is adjusted following the discrete staircase schedule, which reduced the learning rate by half per 200 iterations; the initial learning rate is equal to 0.2, the solver type is “SGD” and the momentum is equal to 0.1. The specific parameter settings of the BPNN model are shown in Table 8.

**Figure 10:**
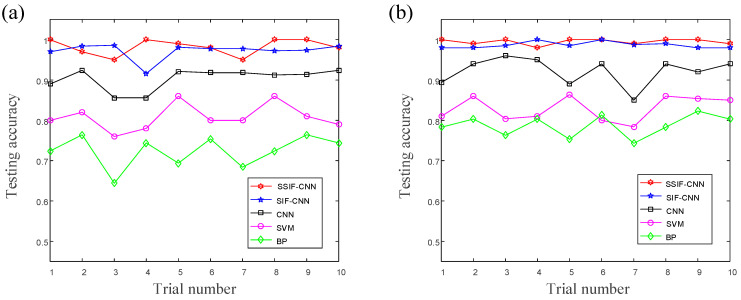
The accuracies of the ten trials: (**a**) training accuracies, and (**b**) testing accuracies.

Due to the random initialization of the weights, the classification performance of the same model is different in the different training processes. Hence, ten repeated trials based on the randomly selected samples strategy are carried out. The training accuracies and testing accuracies of the ten trials are shown in Figure 10. Table 9 shows the classification performance of different models achieved from the ten repeated experiments.

As shown in Figure 10, the training accuracy of the proposed model reaches 100% in six of the ten trials, and the testing accuracy reaches 100% in four of the ten trials. All the accuracies of the proposed model maintain an accuracy level above 95%, which shows that the proposed model has excellent performance not only in high classification accuracy but, also, in classification stability. As illustrated in Table 8, the normal CNN has an average accuracy of training accuracy with 91.8% and test accuracy with 91.6%, and the average training time is about 167.3 s. The average training time of the SIF-CNN is about 192.8 s, the average training accuracy is 99.1% and the average testing accuracy is 98.2%. The average testing accuracy training time of the SSIF-CNN is about 124.2 s, the average training accuracy is 99.5% and the average testing accuracy is 98.6%. The SVM and BPNN have poor classification performances and need more time to complete the model training.

Due to fewer fully connected layer parameters in the SSIF-CNN framework, the model training speed is faster, and test accuracy is slightly improved. The SSIF-CNN not only controls the complexity of the machine learning but, also, has faster convergence speeds and higher identification ratios.

### 4.2. Application Verification

#### 4.2.1. Data Description

**Figure 11:**
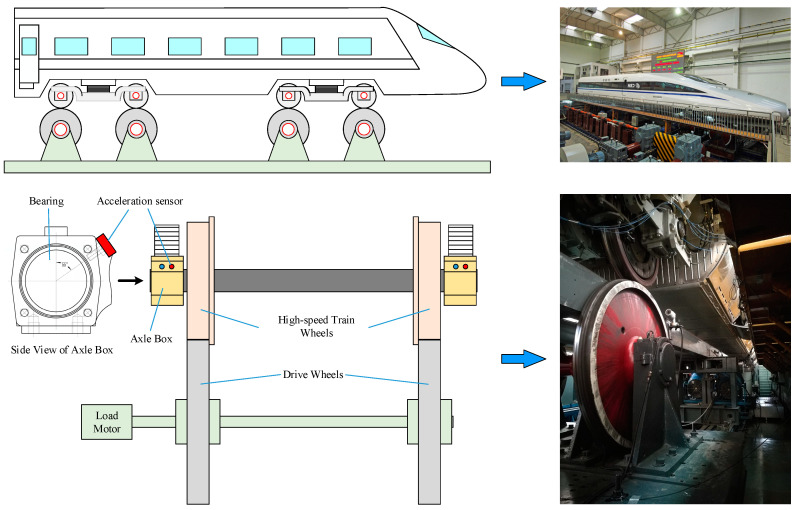
High-speed train rolling test rig.

The axle-box bearing fault data is obtained with a train rolling test rig from the National Engineering Laboratory for High-Speed Trains. As shown in Figure 11, the test rig consists of load motors, drive wheels, acceleration sensors, speed sensors and a national instrument (NI) data acquisition system. The load motor drives the driving wheels to rotate, and the high-speed train wheel, as the driven wheel, is driven to simulate the actual working condition of the high-speed train.

Eight rolling bearings, collected from the locomotive maintenance, are mounted on the test train, and their conditions are listed in Table 10. Several experiments are carried out under different working conditions, and the vibration data is collected with a sample rate of 20 kHz.

The datasets acquired from an experiment running at a speed of 200 km/h are used to identify and classify the axle-box bearing faults. In this experiment, the axle rotation speed is 1233 rpm, and five kinds of defects are included: BF+ORF, IRF+ORF and ORF with three different sizes. Table 11 shows the information of the experimental datasets. Each bearing condition has 200 training samples and 200 testing samples, and each sample contains 2048 data points. The total number of training samples is 1200 (200 × 6), while that of testing samples is also 1200 (200 × 6).

#### 4.2.2. Diagnostic Results

**Figure 12:**
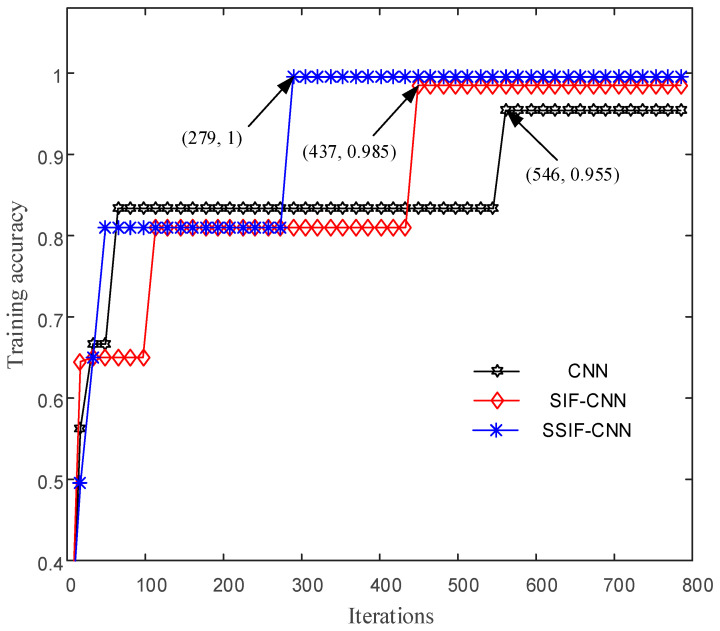
Accuracy of the different CNNs with iterations.

After mixing up the samples, the whole batch of samples is input into the training models for ten repeated experiments, and the results of the first trial are shown in Figure 12. The general CNN will converge after 546 iterations, and the average training accuracy is 95.8%. The accuracy of the SIF-CNN achieves 100% after 392 iterations. The convergence speed of the SSIF-CNN, with 258 iterations, is much faster than the general CNN and SIF-CNN.

The fault classification accuracy specifications of the three models are listed in Table 12 and Table 13 in detail. Table 12 shows the specifications of the classification accuracy of the training samples, while Table 13 shows the same thing for the testing samples. In the CNN training process, the accuracy of bearing conditions 4 and 5 only reaches 93% and 86%, whereas all the accuracies of the SIF-CNN model maintain levels above 92%. The SSIF-CNN classifies all the training samples with an accuracy of 100%. In the testing process, the accuracy of bearing conditions 4 and 5 only reaches 85% and 82% by the CNN, respectively, and the SIF-CNN model has a classification accuracy with at least 90%. The SSIF-CNN classifies all the training samples with an accuracy of 100%.

**Figure 13:**
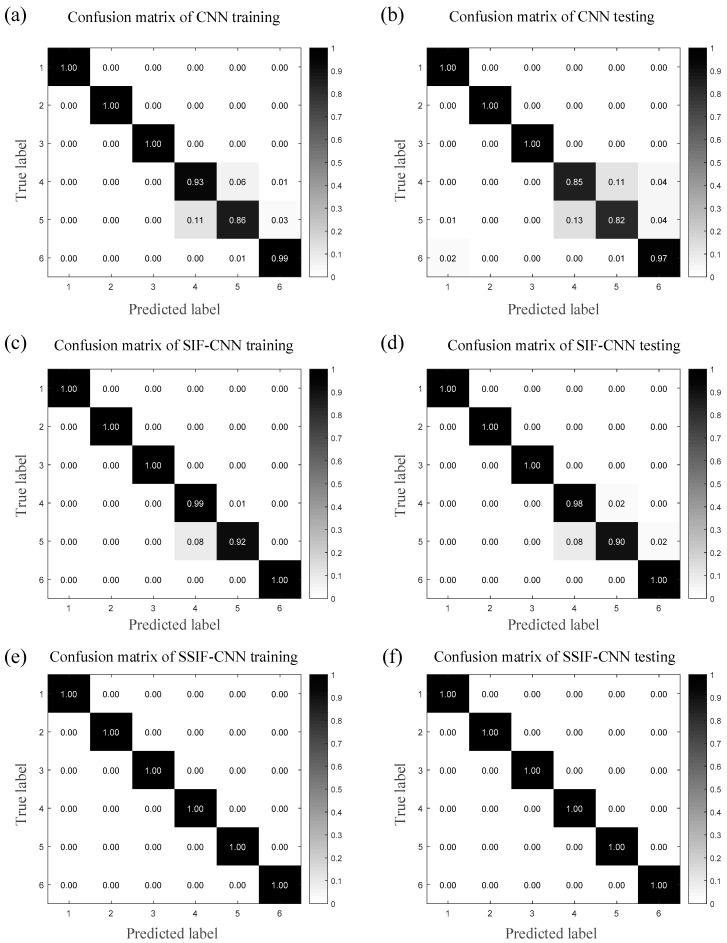
Confusion matrix of the three models for the first trial.

Figure 13 shows that the confusion matrix thoroughly records the diagnosis classification results of the different bearing conditions, including both the classification information and misclassification information. As shown in Figure 13a,b, the CNN fails to classify bearing condition 4 and bearing condition 5. The lowest accuracy happens in condition 5 for training and that of testing happens in condition 5, too. It can be seen from Figure 13c,d, in which the lowest accuracy happens in condition 5 for the SIF-CNN training and that of testing happens in condition 5. As shown in Figure 13e,f, the proposed method can classify all the fault types accurately.

**Figure 14:**
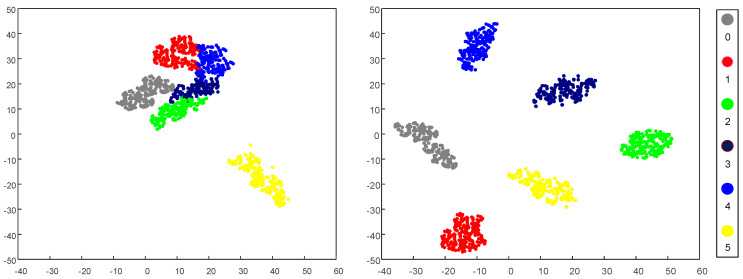
Feature clustering effect diagram: (**a**) raw data and (**b**) SSIF-CNN.

The feature representation of the fully connected layer of the SSIF-CNN is reduced to a two-dimensional distribution by t-SNE. As shown in Figure 14, the features of the different fault types can be clearly classified, which indicates that the SSIF-CNN is an effective approach for high-speed train axle bearing fault classification.

**Figure 15:**
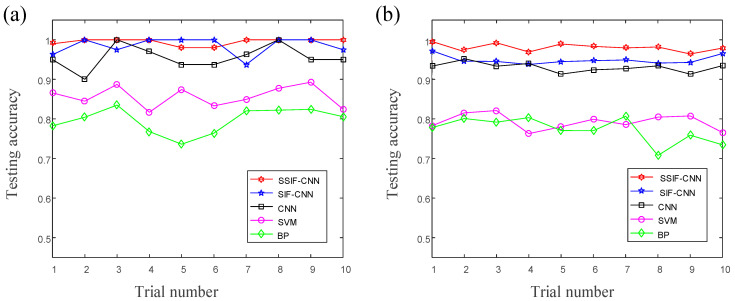
The accuracies of the ten trials: (**a**) training accuracies, and (**b**) testing accuracies.

The training accuracies and testing accuracies of the ten trials are shown in Figure 15. As shown in Figure 15, the training accuracy of the proposed model reaches 100% in seven of the ten trials, and the testing accuracy reaches 100% in three of the ten trials. All the accuracies of the proposed model maintain an accuracy level above 95%, which shows that the proposed model has excellent performance not only in high classification accuracy but, also, in classification stability. The classification performances of the different models, which were achieved from ten repeated experiments with a maximum of 2000 iterations, are listed in Table 14. Due to fewer parameters, the average training speed of the SSIF-CNN, with 64.8 s, is much faster than the general CNN and SIF-CNN. Compared to the result of case 1, the SIF-CNN has more parameters, but the training time is shorter than the general CNN, which shows that fusing shallow information to the fully connected layer can improve the training efficiency.

## 5. Conclusions

For the security and stability of high-speed trains, the failure monitoring of axle-box bearings has stringent reliability and real-time requirements. To address this challenge, we proposed a fault diagnosis method for axle-box bearing of high-speed trains based on a novel CNN model to improve the computational efficiency and the diagnostic accuracy of the fault diagnosis in this paper. The proposed approach takes advantage of the shallow information while reducing the dimension of the parameters of the CNN model to shorten the training time and improve the accuracy of the fault diagnosis. Two case studies are carried out to verify the effectiveness of the proposed model, and the results show that the SSIF-CNN has a higher recognition accuracy and faster convergence speed.

In future works, the axle-box bearing fault diagnosis method based on SSIF-CNN needs further optimization. The sensitive features selection can be carried out to improve the diagnostic efficiency. The construct of SSIF-CNN, such as the layer number and the number of neurons in each layer, needs to be optimized to achieve better adaptability. In addition, the SSIF-CNN-based end-to-end deep-learning method should be applied to detect the axle-box bearing fault using the raw acceleration signal rather than inputting the signal feature.

## Figures and Tables

**Table 1:** List of features.

Symbol	Equation	Description	Symbol	Equation	Description
P1	$\frac{1}{N}\overset{N}{\underset{I-1}{\sum }}x_{i}$	Mean	P16	$\frac{P_{10}}{{\left(\sqrt{P_{8}}\right)}^{4}}$	Coefficient of kurtosis
P2	$\sqrt{\frac{1}{N}\overset{N}{\underset{i=1}{\sum }}x_{i}^{2}}$	Root mean square	P17	$\frac{\sum_{k=1}^{K}s\left(k\right)}{K}$	Mean frequency
P3	${\left(\frac{1}{N}\overset{N}{\underset{i=1}{\sum }}\sqrt{\left|x_{i}\right|}\right)}^{2}$	Square root amplitude	P18	$\frac{\sum_{k=1}^{K}{\left(s\left(k\right)-P_{17}\right)}^{2}}{K}$	Spectral variance
P4	$\frac{1}{N}\overset{N}{\underset{i=1}{\sum }}\left|x_{i}\right|$	Absolute average	P19	$\frac{\sum_{k=1}^{K}{\left(s\left(k\right)-P_{17}\right)}^{3}}{K{\left(\sqrt{P_{18}}\right)}^{3}}$	Spectral skewness
P5	$\max\left(x_{i}\right)$	Maximum	P20	$\frac{\sum_{k=1}^{K}{\left(s\left(k\right)-P_{17}\right)}^{4}}{K{\left(P_{18}\right)}^{2}}$	Spectral kurtosis
P6	$\min\left(x_{i}\right)$	Minimum	P21	$\frac{\sum_{k=1}^{K}s\left(k\right)f_{k}}{\sum_{k=1}^{K}s\left(k\right)}$	Frequency center
P7	$P_{5}-P_{6}$	Peak to peak	P22	$\sqrt{\frac{\sum_{k-1}^{K}{\left(f_{k}-P_{21}\right)}^{2}s\left(k\right)}{\sum_{k=1}^{K}s\left(k\right)}}$	Standard deviation frequency
P8	$\frac{1}{N}\overset{N}{\underset{i=1}{\sum }}{\left(x_{i}-\bar{x}\right)}^{2}$	Variance	P23	$\sqrt{\frac{\sum_{k=1}^{K}{\left(f_{k}\right)}^{2}s\left(k\right)}{\sum_{k=1}^{K}s\left(k\right)}}$	Root mean square frequency
P9	$\frac{\sum_{i=1}^{N}{\left(x_{i}-\bar{x}\right)}^{3}}{\left(N-1\right){\left(\sqrt{P_{8}}\right)}^{3}}$	Skewness	P24	$\sqrt{\frac{\sum_{k=1}^{K}{\left(f_{k}\right)}^{4}s\left(k\right)}{\sum_{k=1}^{K}{\left(f_{k}\right)}^{2}s\left(k\right)}}$	P24–P25 are indicators for main frequency band position
P10	$\frac{\sum_{i=1}^{N}{\left(x_{i}-\bar{x}\right)}^{4}}{\left(N-1\right){\left(\sqrt{P_{8}}\right)}^{4}}$	Kurtosis	P25	$\frac{\sum_{k=1}^{K}{\left(f_{k}\right)}^{2}s\left(k\right)}{\sqrt{\sum_{k=1}^{K}s\left(k\right)\sum_{k=1}^{K}{\left(f_{k}\right)}^{4}s\left(k\right)}}$	
P11	$\frac{P_{2}}{P_{4}}$	Shape factor	P26	$\frac{1}{P_{21}}\sqrt{\frac{\sum_{k=1}^{K}{\left(f_{k}-P_{21}\right)}^{2}s\left(k\right)}{K}}$	P26–P29 are indicators for the dispersion or concentration of the spectrum
P12	$\frac{P_{5}}{P_{2}}$	Crest factor	P27	$\frac{\sum_{k=1}^{K}{\left(f_{k}-P_{21}\right)}^{3}s\left(k\right)}{K{\left(P_{22}\right)}^{3}}$	
P13	$\frac{P_{5}}{P_{4}}$	Impulse factor	P28	$\frac{\sum_{k=1}^{K}{\left(f_{k}-P_{21}\right)}^{4}s\left(k\right)}{K{\left(P_{22}\right)}^{4}}$	
P14	$\frac{P_{5}}{P_{3}}$	Coefficient of variation	P29	$\frac{\sum_{k=1}^{K}\sqrt{f_{k}-P_{21}}s\left(k\right)}{K\sqrt{P_{22}}}$	
P15	$\frac{P_{9}}{{\left(\sqrt{P_{8}}\right)}^{3}}$	Coefficient of skewness			

Note: $x\left(i\right)=\left(x_{1},x_{2},\cdots ,x_{N}\right)$ is the sequence of time domain signal, *N* is the length of the signal, $s\left(k\right)=\left(s_{1},s_{1},\cdots ,s_{K}\right)\;$is the spectrum of signal x(i), *k* is the total number of spectral lines and $f_{k}$ is the frequency of the kth spectral line.

**Table 2:** Specific setting parameters of the convulational neural network (CNN).

Layer	Parameters	Output Size	Activation Function
Input layer	29 feature values	29 × 1	-
Convolutional Layer 1	8 kernels, kernel size: 6 × 1	24 × 1 × 8	ReLU
Convolutional Layer 2	16 kernels, kernel size: 4 × 1	21 × 1 × 16	ReLU
Convolutional Layer 3	32 kernels, kernel size: 2 × 1	20 × 1 × 32	ReLU
Fully connected layer	640 neurons	1 × 640	ReLU
Classifier hidden layer	50 neurons	640 × 50	Sigmoid
Classification layer	*n* neurons	50 × *n*	Softmax

Note: *n* is the number of bearing conditions.

**Table 3:** Specific setting parameters of the shallow information fusion-convolutional neural network (SIF-CNN).

Layer	Parameters	Output Size	Activation Function
Input layer	29 feature values	29 × 1	-
Convolutional Layer 1	8 kernels, kernel size: 6 × 1	24 × 1 × 8	ReLU
Convolutional Layer 2	16 kernels, kernel size: 4 × 1	21 × 1 × 16	ReLU
Convolutional Layer 3	32 kernels, kernel size: 2 × 1	20 × 1 × 32	ReLU
Fully connected layer	1168 neurons	1 × 1168	ReLU
Classifier hidden layer	50 neurons	1168 × 50	Sigmoid
Classification layer	*n* neurons	50 × *n*	Softmax

Note: *n* is the number of bearing conditions.

**Table 4:** Specific setting parameters of the simplified shallow information fusion-convolutional neural network (SSIF-CNN).

Layer	Parameters	Output Size	Activation Function
Input layer	29 feature values	29 × 1	-
Convolutional Layer 1	8 kernels, kernel size: 6 × 1	24 × 1 × 8	ReLU
Global convolution Layer 1	8 kernels, kernel size: 24 × 1	1 × 8	ReLU
Convolutional Layer 2	16 kernels, kernel size: 4 × 1	21 × 1 × 16	ReLU
Global convolution Layer 2	16 kernels, kernel size: 21 × 1	1 × 16	ReLU
Convolutional Layer 3	32 kernels, kernel size: 2 × 1	20 × 1 × 32	ReLU
Global convolution Layer 3	32 kernels, kernel size: 20 × 1	1 × 32	ReLU
Fully connected layer	56 neurons	1 × 56	ReLU
Classifier hidden layer	50 neurons	56 × 50	Sigmoid
Classification layer	*n* neurons	50 × *n*	Softmax

Note: *n* is the number of bearing conditions.

**Table 5:** Description of the sample distribution. IRF: inner race fault, ORF: outer race fault and BF: ball fault.

Bearing Condition	Fault Depth (mm)	Fault Pattern	Size of Training/Testing Sample
Normal	0	0	400/400
IRF	0.18	1	400/400
IRF	0.36	2	400/400
IRF	0.54	3	400/400
IRF	0.72	4	400/400
ORF	0.18	5	400/400
ORF	0.36	6	400/400
ORF	0.54	7	400/400
BF	0.18	8	400/400
BF	0.36	9	400/400
BF	0.54	10	400/400
BF	0.72	11	400/400

**Table 6:** The classification accuracy of the training samples.

Model	Accuracy	Accuracy of Each Bearing Condition											
		0	1	2	3	4	5	6	7	8	9	10	11
CNN	0.8977	1	1	0.935	1	1	1	1	0.52	1	0.9175	0.42	1
SIF-CNN	0.9875	1	1	1	1	1	0.89	1	1	1	0.9675	0.985	1
SSIF-CNN	1	1	1	1	1	1	1	1	1	1	1	1	1

**Table 7:** The classification accuracy of the testing samples.

Model	Accuracy	Accuracy of Each Fault Pattern											
		0	1	2	3	4	5	6	7	8	9	10	11
CNN	0.89	1	1	1	1	1	1	1	0.31	1	1	0.375	1
SIF-CNN	0.978	1	1	0.98	1	1	1	1	1	1	0.945	0.81	1
SSIF-CNN	1	1	1	1	1	1	1	1	1	1	1	1	1

**Table 8:** Specific setting parameters of the back propagation neural network (BPNN).

Layer	Parameters	Output Size	Activation Function
Input layer	29 feature values	29 × 1	-
Hidden layer	50 neurons	29 × 50	Sigmoid
Classification layer	*n* neurons	50 × *n*	Softmax

Note: *n* is the number of bearing conditions.

**Table 9:** Average training time and accuracy of each model. SVM: support vector machine.

Model	Training Time	Training Accuracy	Testing Accuracy
CNN	167.3 s	91.8%	91.6%
SIF-CNN	192.8 s	99.1%	98.2%
SSIF-CNN	124.2 s	99.5%	98.6%
SVM	181.1 s	82.5%	79.8%
BPNN	253.3 s	78.3%	72.6%

**Table 10:** Conditions of the axle-box bearings. IRF: inner race fault, ORF: outer race fault and BF: ball fault.

**Bearing Number**	1	2	3	4	5	6	7	8
**Fault Type**	Normal	ORF	BF+ORF	ORF	Normal	ORF	ORF	IRF+ORF

**Table 11:** Description of the datasets.

Bearing Condition	Fault Pattern	Fault Depth	Size of Training Sample/Testing Sample
Normal	1	-	200/200
BF+ORF	2	-	200/200
IRF+ORF	3	-	200/200
ORF	4	Small	200/200
ORF	5	Medium	200/200
ORF	6	Large	200/200

**Table 12:** The classification accuracy of the training samples.

Model	Accuracy	Accuracy of Each Bearing Condition					
		1	2	3	4	5	6
CNN	0.955	1	1	1	0.93	0.86	0.99
SIF-CNN	0.985	1	1	1	0.99	0.92	1
SSIF-CNN	1	1	1	1	1	1	1

**Table 13:** The classification accuracy of the testing samples.

Model	Accuracy	Accuracy of Each Bearing Condition					
		1	2	3	4	5	6
CNN	0.94	1	1	1	0.85	0.82	0.97
SIF-CNN	0.98	1	1	1	0.98	0.90	1
SSIF-CNN	1	1	1	1	1	1	1

**Table 14:** Training time and accuracy of each model.

Model	Training Time	Training Accuracy	Test Accuracy
CNN	112.4 s	95.5%	93%
SIF-CNN	105.6 s	98.5%	94.5%
SSIF-CNN	64.8 s	99.5%	98%
SVM	119.5 s	86%	79.5%
BP	162.6 s	81%	77.5%
